# Novel paramyxovirus in wild pinnipeds, Brazil

**DOI:** 10.1007/s11259-025-10799-5

**Published:** 2025-07-01

**Authors:** Samira Costa-Silva, Ana Carolina Ewbank, Aricia Duarte-Benvenuto, Carlos Sacristán, Rodrigo M. Soares, Angélica M. Sánchez-Sarmiento, Natália Silvestre-Perez, Pedro Volkmer Castilho, Cristiane K. M. Kolesnikovas, Caroline Freitas Pessi, Rafael Sardinha Murro, Carla B. Barbosa, Raquel B. Ferioli, José L. Catão-Dias, Lara B. Keid

**Affiliations:** 1 Faculdade de Medicina Veterinária e Zootecnia, Universidade de São Paulo, São Paulo, 05508-270 SP Brazil; 2 Centro de Investigación en Sanidad Animal (CISA-INIA), CSIC, Valdeolmos, 28130 Spain; 3 Faculdade de Zootecnia e Engenharia de Alimentos, Universidade de São Paulo, Pirassununga, São Paulo 13635-900 Brazil; 4 Instituto Argonauta para Conservação Costeira e Marinha, Ubatuba, São Paulo 11680-000 Brazil; 5 Universidade do Estado de Santa Catarina-UDESC, Laguna, Santa Catarina 13635-900 Brazil; 6 Associação R3 Animal, Florianópolis, Santa Catarina, 88061-500 Brazil; 7 Instituto de Pesquisas de Cananéia, Cananéia, São Paulo 11990-000 Brazil

**Keywords:** Coronavirus, Emerging virus, Fur seal, *Jeilongvirus*, Marine mammals, *Paramyxoviridae*

## Abstract

Most emerging zoonotic viral infections originate in wildlife. The *Paramyxoviridae* and *Coronaviridae* families are important in terms of zoonotic potential and diversity of host susceptibility. To date, the only paramyxoviruses described in pinnipeds are within the genus *Morbillivirus*. An alphacoronavirus has been reported in pinnipeds. Herein, we surveyed for *Paramyxoviridae* and *Coronaviridae* in 46 animals of four pinniped species stranded along the coasts of Santa Catarina and São Paulo states, Brazil, between 2016 and 2022. A novel paramyxovirus (family *Paramyxoviridae*) was detected in an Antarctic fur seal (*Arctocephalus gazella*) and in two South American fur seals (*Arctocephalus australis*). The sequences presented with less than 83% amino acid identity to the closest published paramyxovirus sequences, amplified in *Microchiroptera* sp. in Vietnam and in Asiatic yellow bats (*Scotophilus kuhlii*) in Cambodia. Phylogenetic analysis of the paramyxovirus sequences clustered into the genus *Jeilongvirus*. Gross and histopathological analyses did not identify any related lesions and the viral zoonotic potential is unknown. None of the animals were PCR-positive to coronavirus. To our knowledge, this is the first report of paramyxovirus infection in pinnipeds in Brazil and the first paramyxovirus not classified as *Morbillivirus* in seals, sea lions and walruses. To better understand the natural history of this novel virus, future pathological, serological, and molecular studies based on pan-*Paramyxoviridae* RT-PCRs are recommended.

**Supplementary Information:**

The online version contains supplementary material available at 10.1007/s11259-025-10799-5.

## Introduction

RNA viruses, particularly those from families *Paramyxoviridae* and *Coronaviridae*, are infectious agents with zoonotic potential (Cox and Plemper 2017; V’kovski et al. 2021). *Paramyxoviridae* (order *Mononegavirales*) members have linear single-stranded negative-sense RNA and can infect a wide range of vertebrates (Cox and Plemper 2017). The subfamily *Orthoparamyxovirinae* includes the *Morbillivirus* and *Henipavirus* genera, which comprise species able to cause severe disease in humans (Qawiem et al. 2022), as well as lesser known genera, such as the *Jeilongvirus* (Zhu et al. 2022). Members of the *Jeilongvirus* have been reported in wild bats, rodents, hedgehogs, and in domestic, feral and wild felids; this genus has been associated with kidney, heart, and liver lesions, and was detected in anal swabs, urine, heart, liver, kidney, and spleen samples (Maganga et al. 2014; Sakaguchi et al. 2020; Sieg et al. 2020; Zhu et al. 2022). The subfamily *Orthocoronavirinae* (family *Coronaviridae*, order *Nidovirales*) comprises four genera (*Alpha-*, *Beta-*, *Gamma-* and *Deltacoronavirus*) of linear single-stranded positive-sense RNA viruses (V’kovski et al. 2021). Infections with coronaviruses can cause disease in a wide range of host species, including humans, as reported for *Middle East respiratory syndrome coronavirus* and *Severe acute respiratory syndrome-related coronavirus* (V’kovski et al. 2021).

Pinnipeds (i.e., seals, fur seals, sea lions and walruses [*Odobenus rosmarus*]) are susceptible to several infectious agents, including zoonotic, emergent and reemergent viruses (Duignan et al. 2018). Morbilliviruses like phocine distemper virus, canine distemper virus and cetacean morbillivirus were described in these mammals, often causing high morbidity and mass mortality events in the northern hemisphere, especially in juvenile animals (Duignan et al. 2014). In pinnipeds, clinical signs associated with morbillivirus infection include nasal discharge, dyspnea, impaired swimming, head tremors, and convulsions (Duignan et al. 2014). Conversely, the only coronavirus reported in pinnipeds is an alphacoronavirus in harbor seals (*Phoca vitulina*) with pneumonia, on the Pacific coast of the USA (Nollens et al. 2010). To date, there is little information regarding paramyxoviruses and coronaviruses infections in pinnipeds of the southern hemisphere, including Brazil. However, despite the absence of reproductive colonies in the country, several pinniped species arrive year-round to the Brazilian coast following the Malvinas current (Duarte-Benvenuto et al. 2024). The goal of this study was to survey the occurrence of viruses within the families *Paramyxoviridae* and *Coronaviridae* in pinnipeds that stranded in São Paulo and Santa Catarina states, Brazil, between 2016 and 2022.

## Materials and methods

### Samples

We analyzed 46 pinnipeds of the species South American fur seal (*Arctocephalus australis*, *n* = 35), subantarctic fur seal (*Arctocephalus tropicalis*, *n* = 5), Antarctic fur seal (*Arctocephalus gazella*, *n* = 4), *Arctocephalus* sp. (*n* = 1), and South American sea lion (*Otaria flavescens*, *n* = 1) that stranded in Santa Catarina and São Paulo states (southeastern Brazil), between 2016 and 2022 (Supplementary Table 1). Overall, 19 individuals were found dead (code 2 or 3), 12 stranded alive and died before rescue (code 2), and 15 animals stranded alive, of which 13 died during treatment and two were released. The decomposition status (code 2 to code 5) of the dead pinnipeds was classified based on Geraci and Lounsbury (2005). Age class (fetus, calf, juvenile and adult) was established based on total body length (Jefferson et al. 2008). Blood, oral swabs, and rectal swabs collected from two live-stranded individuals that were subsequently released were frozen at −80 ºC. Multiple tissues from the carcasses of the remaining 44 individuals were sampled for molecular (frozen at −20 ºC and − 80 ºC) and histopathological analyses (10% buffered formalin), including brain, lung, liver, spleen, lymph nodes, intestine, and kidney (Supplementary Table 2). These tissues were selected to cover potential viral tropism.

All individuals were found during the year-round daily monitoring program performed by the Santos Basin Beach Monitoring Project (Projeto de Monitoramento de Praias da Bacia de Santos - PMP-BS), funded by Petrobrás, and licensed by the Brazilian Institute of the Environment and Renewable Natural Resources (IBAMA) of the Brazilian Ministry of Environment (ABIO Nº 640/2015.

### Molecular techniques

**Fig. 1:**
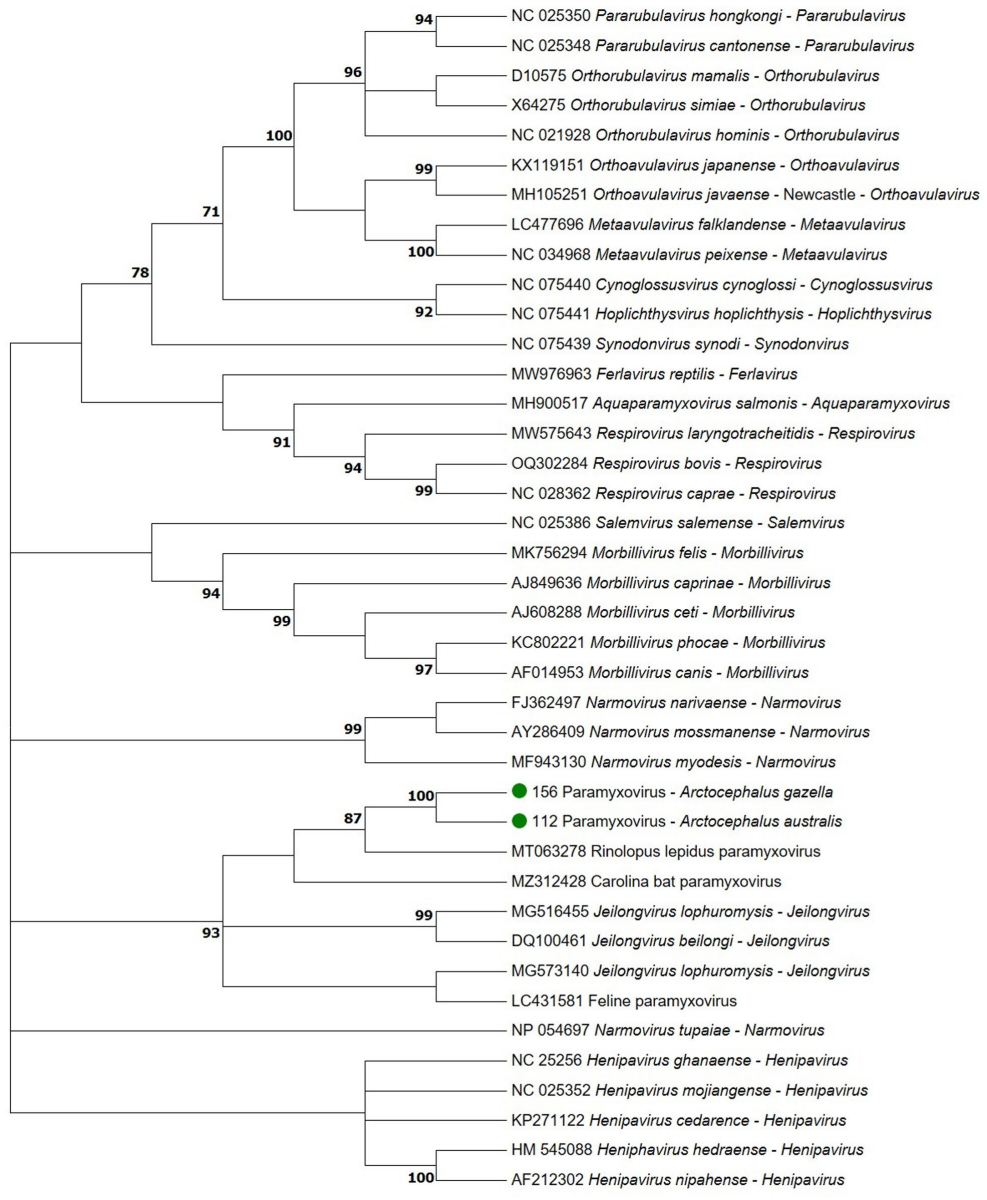
Consensus maximum likelihood phylogenetic tree based on Le Gascuel model with inversions, gamma distribution and invariant sites of the alignment of (1) the amino acid paramyxovirus *RNA-dependent RNA polymerase* sequences retrieved in two South American fur seals (*Arctocephalus australis*, ID 112/23; ID 118/23) and an Antarctic fur seal (*Arctocephalus gazella*, ID 156/21) (green dots); and (2) representative paramyxovirus sequences of viral species of the same gene recognized by the International Committee on Taxonomy of Viruses. The bootstrap consensus tree inferred from 1000 replicates represents the evolutionary history of the analyzed taxa. ProtTest test was used to select the evolutionary model for the phylogram. Bootstrap values < 70 were omitted

Frozen samples were mechanically homogenized using sterilized scalpel blades and Petri dishes (Supplementary Table 1). Subsequently, total RNA was extracted from selected frozen tissue samples (Supplementary Table 1) using TRIzol-LS (Life Technologies Corporation, CA, USA). Reverse transcription was performed using random primers, oligo dT, and Moloney Murine leukemia virus reverse transcriptase (iScript™ cDNA Synthesis Kit, Bio-Rad, CA, USA). To screen for *Paramyxoviridae* and *Coronaviridae* families, the RNA-dependent RNA polymerase (RdRp) gene was amplified using a nested PCR (Tong et al. 2008), and a semi-nested PCR (Holbrook et al. 2021), respectively. Amplicons were visualized in 2% agarose gel electrophoresis, and the obtained amplicons of the expected size were purified using PureLink™ Quick Gel Extraction Kit (Life Technologies Corporation). Both strands were directly sequenced by Sanger using the ABI PRISM BigDye^®^ Terminator v3.1 kit (Ready Reaction Cycle Sequencing, Applied Biosystems, Foster City, USA). The retrieved sequences were assembled using MEGA 7 (Kumar et al. 2016), and the primers were edited out. After BLASTn and BLASTp searches of the consensus sequences on NCBI (https://blast.ncbi.nlm.nih.gov/Blast.cgi) and subsequent ClustalW alignment in MEGA 7, the percentage of nucleotide (nt) and amino acid (aa) identities among the obtained consensus sequences and the closest ones from GenBank were calculated based on p-distance ([1– p distance]*100]. A paramyxovirus maximum likelihood amino acid tree of RdRp gene was built using MEGA 7 with 1000 bootstrap replicates, selecting the longest unique amino acid sequence types retrieved in our study and representative viruses within the family *Paramyxovidirae* (Fig. 1). The ProtTest 3 test program was used to select the evolutionary model for the phylogram (Darriba et al. 2011).

## Results

All animals were negative for coronavirus. Three of 46 pinnipeds (6.5%) were positive by pan-paramyxovirus RT-PCR; an *A. gazella* (ID 156/21) and two *A. australis* (IDs 112/23 and 118/23) (Supplementary Table 1). The *A. gazella* tested positive in brainstem, spinal cord and kidney; its clinical signs and treatment were reported by Duarte-Benvenuto et al. (2024). The two emaciated *A. australis* were found dead (code 2 and had viral RNA detected in kidney samples. Gross and histological findings, biologic, epidemiologic and molecular data of positive individuals are described in Table 1.

**Table 1:** Biologic and molecular data of the paramyxovirus-positive pinnipeds stranded at the Santa Catarina and São Paulo Coasts (Brazil), between 2016 and 2022, analyzed in this study. J = Juvenile. M = Male. F = Female

ID	Species	Age class	Sex	Year and stranding location	L gene sequence length (bp)	GenBank Accession Number	nt Identity	Closest available sequence	Species in which the closest sequence was detected	Gross findings	Histological findings
112/23	*Arctocephalus australis*	J	M	2018, Laguna-SC	530	OR867078	81.8%	OQ715608	*Rhinolopus sinicus*	Scapular hematoma, pale tissues and pulmonary edema	**Lymph node**: diffuse mild lymphoid hyperplasia with mild lymphocytosis. **Lung**: mild neutrophilic lymphadenitis, mild diffuse bronchointerstitial lymphoplasmacytic pneumonia, edema and congestion.
118/23	*Arctocephalus australis*	J	F	2017, Imbituba-SC	512	OR867079	82.8%	OQ715608	*R. sinicus*	Good body condition. Focal circular well-defined ulcerative skin lesions of 1–3 cm in diameter on the ventral aspect of the pectoral fins. Moderate to marked diffuse pulmonary congestion. Mild diffuse brain congestion. Mild to moderate multifocal to coalescent gastritis.	**Heart**: mild multifocal cardiac myolysis. **Lymph node**: moderate lymphoid hyperplasia with expansion of germinal center and mild lymphocytosis. Rare granulocytes on paracortical zone. **Kidney**: mild to moderate diffuse congestion. **Liver**: mild focal predominantly midzonal mixed hepatitis. **Spleen**: mild to moderate hyperplasia of periarteriolar lymphoid sheaths with mild lymphocytosis. Mild diffuse acute splenitis. **Lung**: moderate to marked diffuse congestion and mild to moderate diffuse edema. Presence of granulated material in alveoli. Focal intravascular bacterial emboli. Moderate presence of coccobacilli bacteria in subpleural region. **Tongue**: mild multifocal mixed sub-epithelial glossitis. **Lymph node**: mild to moderate diffuse mixed lymphadenitis. Moderate diffuse lymphoid hyperplasia and lymphocytosis on germinal centers. **NSFO**: adrenal gland (partially autolyzed), trachea, intestines, bladder, pancreas, skin, and skeleton muscle.
156/21	*Arctocephalus gazella*	J	M	2021, São Paulo	530	OR867080 (of kidney and spinal cord) and OR867081 (of brainstem)	82.8%	OQ715608	*R. sinicus*	Duarte-Benvenuto et al. 2024	Duarte-Benvenuto et al. 2024.

We found four sequence types: two in the *A. gazella* (also highly similar [99.6 nt identity, 100 aa identity] between them) and one in each *A. australis* (highly similar between them [98.3% nt identity; 99.4% aa identity]). When compared, the two sequence types from *A. australis* presented high nt (98.7% to ID 156/21 brainstem, 99.2% to ID 156/21 spinal cord and kidney) and aa identities (98.8% for ID 112/23, 99.4% for ID118/23) with the *A. gazella* sequences. The nt and aa substitutions among all the pinniped paramyxoviruses detected in Brazil are displayed in Supplementary Table 2. Our sequences have the highest nt identities (81.8–82.8%) with a paramyxovirus identified in a Chinese horseshoe bat (*Rhinolopus sinicus*) in China (OQ715608). The highest aa identities (ranging from 81.8 to 82.4%) are with paramyxovirus sequences retrieved in a *Microchiroptera* sp. in Vietnam and in Asiatic yellow bats (*Scotophilus kuhlii*) in Cambodia (Supplementary Table 2).

## Discussion

The phylogram correctly classified most of the members of the *Paramyxoviridae* family, with strong statistical support (Cox and Plemper 2017). A statistically valid clade was formed by the pinniped sequences, the paramyxovirus sequence of bats, and those within the genus *Jeilongvirus*. The findings are included in the phylogram (Fig. 1). A limitation of the study was that we were not able to obtain the complete RdRp sequence; thus, further characterization of additional molecular markers, genome organization, used receptors, and host range are required in order to definitively classify this recently discovered virus within the genus *Jeilongvirus* (Cox and Plemper 2017).

Our sequence similarity and phylogenetic analyses indicate that the novel sequences are closer to *Jeilongvirus*, a viral genus recently described in carnivores– domestic cats in Japan and guignas (*Leopardus guigna*) in Chile (Sieg et al. 2020), among other mammals (Zhu et al. 2022). Although the paramyxovirus sequences were detected in the kidneys of all positive individuals, and in the brainstem and spinal cord of one animal, compatible histopathologic lesions were not observed in these tissues (Vanmechelen et al. 2020). The detection of a novel paramyxovirus in three individuals of two distinct species suggests exposure in breeding or otherwise shared areas (e.g., during migration). Additionally, all three pinnipeds were juveniles, suggesting decreased maternal passive immunity as a risk factor.

## Conclusion

We described a novel paramyxovirus in pinnipeds in Brazil. Although paramyxoviruses apparently can switch between different hosts, the public health risk of this novel virus has not yet been determined. Further studies, including viral isolation, whole genome sequencing, from a larger number of animals and a wider geographical area are necessary to elucidate the taxonomy, pathogenicity and epidemiology of this virus. The authors recommend future molecular and serological studies of the *Paramyxoviridae* family in pinnipeds.

## Electronic supplementary material

Below is the link to the electronic supplementary material.

Supplementary Material 1

## Data Availability

All data generated or analyzed during this research are included in the manuscript. The nucleotide sequences obtained in this research were submitted to GenBank under accession numbers OR867078-OR867081.
