# Supplementary material for: Novel paramyxovirus in wild pinnipeds, Brazil
Source: Vet Res Commun. 2025 Jul 1;49(4):242. doi: 10.1007/s11259-025-10799-5 (PMC12213916; doi:10.1007/s11259-025-10799-5)
Supplement: Supplementary file 1 — Supplementary Material 1 [file 11259_2025_10799_MOESM1_ESM.docx]

**Supplementary figure 1.** Spatial location of the paramyxovirus-positive animals along the Brazilian coast. Location of the two South American fur seals (*Arctocephalus australis*) stranded in Santa Catarina state (SC), in 2017 and 2019 (pink dots), and of the Antarctic fur seal (*Arctocephalus gazella*) stranded in São Paulo state (SP), in 2021 (blue dots).


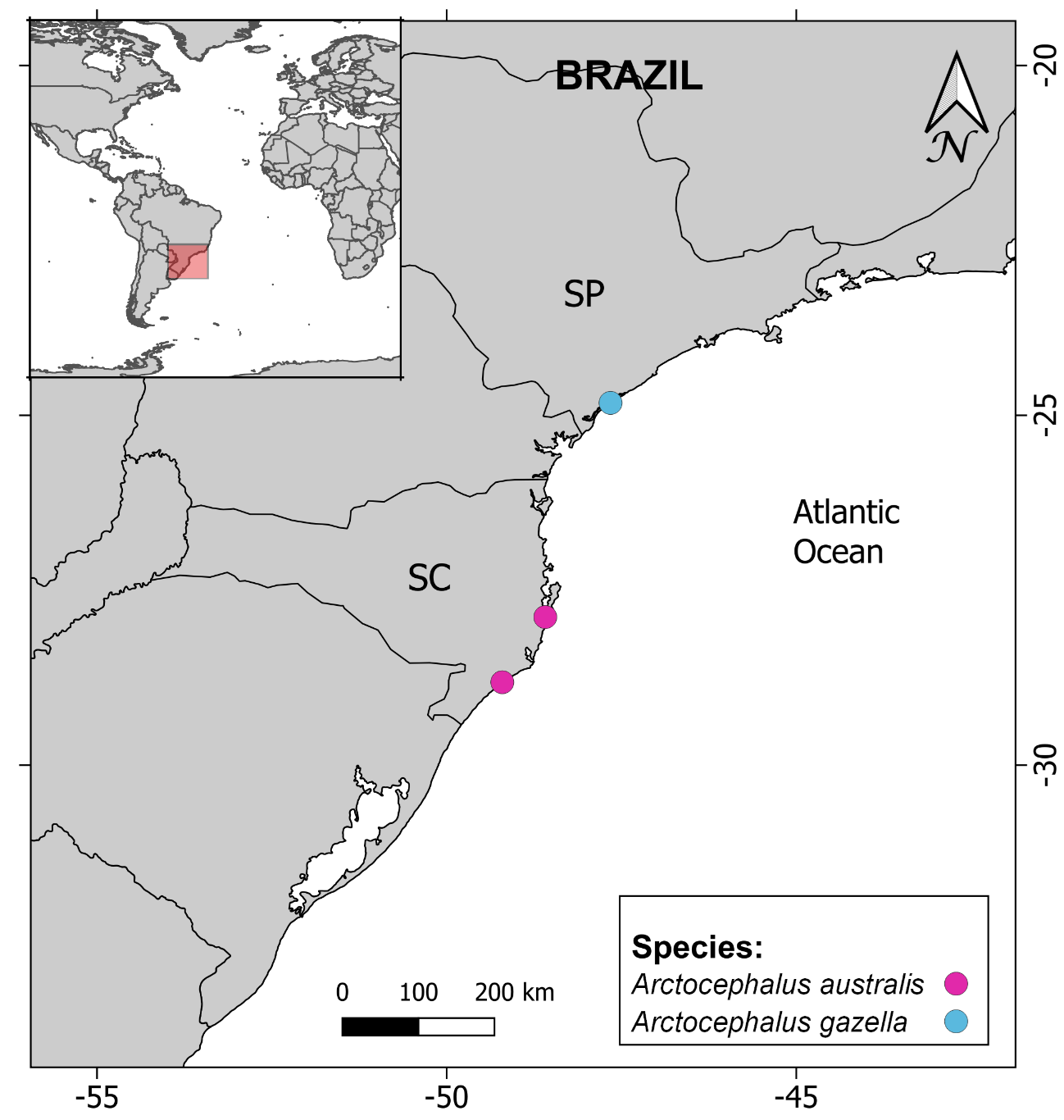


**Supplementary Table 1**. Biological (age class, sex) and epidemiological parameters (location and date of stranding), and tested organs of the pinnipeds stranded in the states of São Paulo (SP) and Santa Catarina (SC), Brazil, between 2016 and 2022, tested herein for paramyxovirus and coronavirus by nested PCR.

| Case number | Species | Body condition | Sex | Age class | Stranded | Local of stranding | Tissues tested for paramyxovirus by PCR* |
| --- | --- | --- | --- | --- | --- | --- | --- |
| 13/23 | *Arctocephalus tropicalis* | Poor | Male | Juvenile | Aug 30, 2021 | Laguna, SC* | Kidney, lung, mesenteric lymph node and spinal cord |
| 28/23 | *Arctocephalus tropicalis* | Poor | Male | Juvenile | Jul 25, 2021 | Florianópolis, SC | Brainstem, lung, kidney, mesenteric lymph node, and spinal cord |
| 30/23 | *Arctocephalus tropicalis* | Poor | Female | Adult | Aug 30, 2020 | Cananéia, SP** | Brainstem, lung, kidney, and mesenteric lymph node |
| 79/20 | *Arctocephalus tropicalis* | Poor | Male | Adult | Jul 21, 2020 | Florianópolis, SC | Blood, oral swab and rectal swab |
| 31/23 | *Arctocephalus tropicalis* | Poor | Female | Adult | Jul 15, 2020 | Cananéia, SP | Brainstem, lung, kidney, mesenteric lymph node, and spinal cord |
| 14/23 | *Arctocephalus australis* | Poor | Female | Juvenile | Aug 6, 2020 | Laguna, SC | Lung, mesenteric lymph node, and prescapular lymph node |
| 15/23 | *Arctocephalus australis* | Poor | Female | Calf | Sep 19, 2020 | Laguna, SC | Kidney, lung, and prescapular lymph node |
| 16/23 | *Arctocephalus australis* | Poor | Female | Juvenile | Sep 28, 2019 | Laguna, SC | Brainstem, lung, mesenteric lymph node, and spinal cord |
| 17/23 | *Arctocephalus australis* | Cachectic | Female | Juvenile | Jun 14, 2018 | Laguna, SC | Brain, lung, mesenteric lymph node, and spinal cord |
| 18/23 | *Arctocephalus australis* | Cachectic | Female | Juvenile | Jul 14, 2018 | Laguna, SC | cerebrum, lung, kidney, meninges, mesenteric lymph node, and spinal cord |
| 19/23 | *Arctocephalus australis* | Cachectic | Male | Juvenile | Jul 7, 2020 | Laguna, SC | Kidney, lung, mesenteric lymph node, and spinal cord |
| 20/23 | *Arctocephalus australis* | Cachectic | Female | Juvenile | Jul 6, 2018 | Laguna, SC | Cerebrum, lung, kidney, meninges, mesenteric lymph node, and spinal cord |
| 21/23 | *Arctocephalus australis* | Cachectic | Male | Calf | Jul 16, 2020 | Laguna, SC | Lung, mesenteric lymph node, and prescapular lymph node |
| 22/23 | *Arctocephalus australis* | Good | Female | Juvenile | Jun 30, 2018 | Laguna-SC | Cerebrum, kidney, mesenteric lymph node, pulmonary lymph node, and spinal cord |
| 23/23 | *Arctocephalus australis* | Good | Female | Juvenile | Aug 22, 2019 | Laguna, SC | Lung, kidney, mesenteric lymph node, and prescapular lymph node |
| 24/23 | *Arctocephalus australis* | Cachectic | Male | Calf | Oct 4, 2021 | Florianópolis, SC | Brainstem, lung, mesenteric lymph node, and spinal cord |
| 25/23 | *Arctocephalus australis* | Poor | Female | Juvenile | Sep 4, 2020 | Laguna, SC | Brainstem, lung, kidney, and spinal cord |
| 26/23 | *Arctocephalus australis* | Cachectic | Female | Juvenile | Aug 23, 2020 | Laguna, SC | Brainstem, lung, kidney, mesenteric lymph node, and spinal cord |
| 27/23 | *Arctocephalus australis* | Poor | Male | Calf | Aug 31, 2020 | Florianópolis, SC | Brainstem, lung, kidney, mesenteric lymph node, and spinal cord |
| 29/23 | *Arctocephalus australis* | Cachectic | Male | Juvenile | Jul 6, 2022 | São Paulo, SP | Brainstem, lung, kidney, mesenteric lymph node, and spinal cord |
| 30/23 | *Arctocephalus australis* | Cachectic | Female | Juvenile | Oct 11, 2021 | São Paulo-SP | Brainstem, lung, kidney, mesenteric lymph node, and spinal cord |
| 36/21 | *Arctocephalus australis* | Cachectic | Male | Juvenile | Oct 15 2020 | Florianópolis, SC | Brainstem, kidney, lung, and spinal cord |
| 30/21 | *Arctocephalus australis* | Poor | Male | Juvenile | Sep 25, 2020 | Florianópolis, SC | Brainstem, kidney, lung, and spinal cord |
| 32/21 | *Arctocephalus australis* | Poor | Male | Juvenile | Oct 18, 2020 | Florianópolis, SC | Brainstem, kidney, lung, and spinal cord |
| 26/21 | *Arctocephalus australis* | Good | Male | Juvenile | Nov 19, 2020 | Imbituba, SC | Brainstem, kidney, lung, and spinal cord |
| 100/23 | *Arctocephalus australis* | Cachectic | Male | Juvenile | Aug 2, 2020 | Laguna, SC | Heart, kidney, lung, liver, prescapular lymph node, spleen, and stomach |
| 102/23 | *Arctocephalus australis* | Poor | Female | Calf | Nov 20, 2021 | Imbituba, SC | Kidney, lung, prescapular lymph node, and spleen |
| 103/23 | *Arctocephalus australis* | Poor | Male | Juvenile | Aug 3, 2020 | Laguna, SC | Kidney, lung, mesenteric lymph node, spleen, and prescapular lymph node |
| 104/23 | *Arctocephalus australis* | Poor | Female | Juvenile | Sep 7, 2020 | Laguna, SC | heart, kidney, lung, mesenteric lymph node, spleen and stomach |
| 111/23 | *Arctocephalus australis* | Poor | Male | Juvenile | Sep 3, 2020 | Laguna, SC | kidney, lung, mesenteric lymph node, spleen, and stomach |
| 112/23 | *Arctocephalus australis* | Poor | Male | Juvenile | Aug 8, 2018 | Laguna, SC | Heart, **kidney (+)**, liver, lung, lymph node spleen, , and prescapular lymph node |
| 113/23 | *Arctocephalus australis* | Cachectic | Male | Juvenile | Aug 17, 2019 | Laguna, SC | Heart, kidney, liver, lung, lymph node, spleen, and stomach |
| 114/23 | *Arctocephalus australis* | Good | Female | Juvenile | Aug 16, 2019 | Imbituba, SC | Heart, kidney, liver, lung, spleen, and stomach |
| 115/23 | *Arctocephalus australis* | Good | Female | Juvenile | Jul 2, 2018 | Imbituba, SC | Cerebrum, kidney, lung, meninges, mesenteric lymph node, pulmonary lymph node, and stomach |
| 116/23 | *Arctocephalus australis* | Cachectic | Female | Juvenile | Aug 9, 2016 | Laguna, SC | Heart, kidney, liver, lung, mesenteric lymph node, and prescapular lymph node |
| 117/23 | *Arctocephalus australis* | Poor | Male | Juvenile | Sept 7, 2017 | Laguna, SC | Cerebrum, heart, kidney, liver, lung, mesenteric lymph node, spleen, and stomach |
| 118/23 | *Arctocephalus australis* | Good | Female | Juvenile | Aug 18, 2017 | Imbituba, SC | **Kidney (+)**, liver, lung, mesenteric lymph node, spleen, and stomach |
| 119/23 | *Arctocephalus australis* | Cachectic | Male | Juvenile | Jun 21, 2017 | Laguna, SC | Cerebrum, kidney, lung, liver, mesenteric lymph node, spleen, and stomach |
| 120/23 | *Arctocephalus australis* | Poor | Male | Juvenile | Jun 22, 2017 | Laguna, SC | Cerebrum, kidney, liver, lung, spleen, and stomach |
| 121/23 | *Arctocephalus australis* | Poor | Male | Juvenile | Aug 29, 2017 | Laguna, SC | Heart, kidney, lung , mesenteric lymph node, spleen, and stomach |
| 24/21 | *Arctocephalus* sp. | Poor | Female | Adult | Jul 29, 2020 | Florianópolis, SC | Blood, oral swab and rectal swab |
| 158/23 | *Arctocephalus gazella* | Poor | Male | Juvenile | Aug 19, 2021 | Florianópolis, SC | Brainstem, kidney, lung, mesenteric lymph node, and spinal cord |
| 03/22 | *Arctocephalus gazella* | Poor | Male | Juvenile | Aug 27, 2021 | Ubatuba, SP | Brainstem, kidney, lung, mesenteric lymph node, and spinal cord |
| 156/21 | *Arctocephalus gazella* | Cachectic | Male | Juvenile | Aug 14, 2021 | Cananéia, SP | **brainstem (+)**, **kidney** (+), lung, mesenteric lymph node, prescapular lymph node, pulmonary lymph node, **spinal cord (+)**, and spleen |
| 157/21 | *Arctocephalus gazella* | Poor | Male | Juvenile | Aug 8, 2021 | Florianópolis, SC | Brainstem, kidney, lung, and spinal cord |
| 04/22 | *Otaria flavescens* | Cachectic | Male | Adult | Apr 14 2021 | Florianópolis, SC | Brainstem, kidney, lung, prescapular lymph node, pulmonary lymph node, and spinal cord |

*Paramyxovirus-positive tissues are marked in bold. *SC: Santa Catarina state, **SP: São Paulo state.

**Supplementary Table 2.** Substitutions observed in the *RNA-dependent RNA polymerase* gene nucleotide and amino acid sequences among all the pinniped paramyxoviruses detected in Brazil, and percentage of similarity between each of them and with the closest paramyxovirus sequences from GenBank, according to BLASTN search and subsequent p-distance analyses.

| **Case ID** | **Year of stranding/ Data of collection** | **Pinniped species** | **GenBank accession nº** | **Nucleotide (nt) sequence size (base pair)** | **Position of nt substitutions related to 112/23 (OR867078) and observed substitutions** | **Amino acid (aa) sequence size** | **Position of aa substitutions related to 112/23 (OR867078)** | **Percentage nt similarity to the closest sequences from GenBank/ENA/DDBJ database** | **Percentage of aa similarity to the closest sequences from GenBank/ENA/DDBJ database** |
| --- | --- | --- | --- | --- | --- | --- | --- | --- | --- |
| 112/23 | 2018 | *Arctocephalus australis* | OR867078 | 530 | - | 176 | - | 81.8% to a paramyxovirus sequence of a  Chinese horseshoe bat (*Rhinolopus sinicus*) of China (OQ715608) | 81.2% to paramyxovirus sequences of a *Microchiroptera* sp. of Vietnam (KP963925) and in lesser Asiatic yellow bats (*Scotophilus kuhlii*) of Cambodia (MT063349 and MT063663) |
| 118/23 | 2017 | *Arctocephalus australis* | OR867079 | 512 | 188 (T/C),  268 (C/T),  304 (C/T),  313 (A/G),  350 (C/T),  352 (A/G),  382 (A/C),  421 (A/G),  436 (A/G),  488 (G/A) | 169 | 163 (V/I) | 82.8% to a paramyxovirus sequence of a  Chinese horseshoe bat of China (OQ715608) | 81.8% to paramyxovirus sequences of a *Microchiroptera* sp. of Vietnam (KP963925) and lesser Asiatic yellow bats (*Scotophilus kuhlii*) of Cambodia (MT063514, MT063349 and MT063663) |
| 156/21 | 2021 | *Arctocephalus gazella* | OR867080  (obtained in kidney and spinal cord) | 530 | 286 (C/T),  313 (A/G),  432 (A/G),  436 (A/G),  488 (G/A) | 176 | 144 (Q/R), 163 (V/I) | 82.8% to a paramyxovirus sequence of a  Chinese horseshoe bat of China (OQ715608) | When the two *A. australis* paramyxovirus nucleotide sequence types were translated to amino acids, the deduced amino acid sequences were identical. They presented 81.8% aa identity to paramyxovirus sequences retrieved in *Microchiroptera* sp. of Vietnam (KP963925) and in lesser Asiatic yellow bats (*Scotophilus kuhlii*) of Cambodia (MT063349 and MT063663) |
|  |  |  | OR867081 (obtained in brainstem) | 530 | 139 (C/T),  286 (C/T),  313 (A/G),  432 (A/G),  436 (A/G),  488 (G/A) | 176 | 144 (Q/R),  163 (V/I) | 82.8% to a paramyxovirus sequence of a  Chinese horseshoe bat of China (OQ715608) |  |
